# Supplementary figures and images for: Privy by the Bay: Emerging hotspot analysis of 311 reports of human/animal waste near San Francisco Pit Stop locations, 2009–2022
Source: PLoS One. 2025 Aug 14;20(8):e0327795. doi: 10.1371/journal.pone.0327795 (PMC12352649; doi:10.1371/journal.pone.0327795)

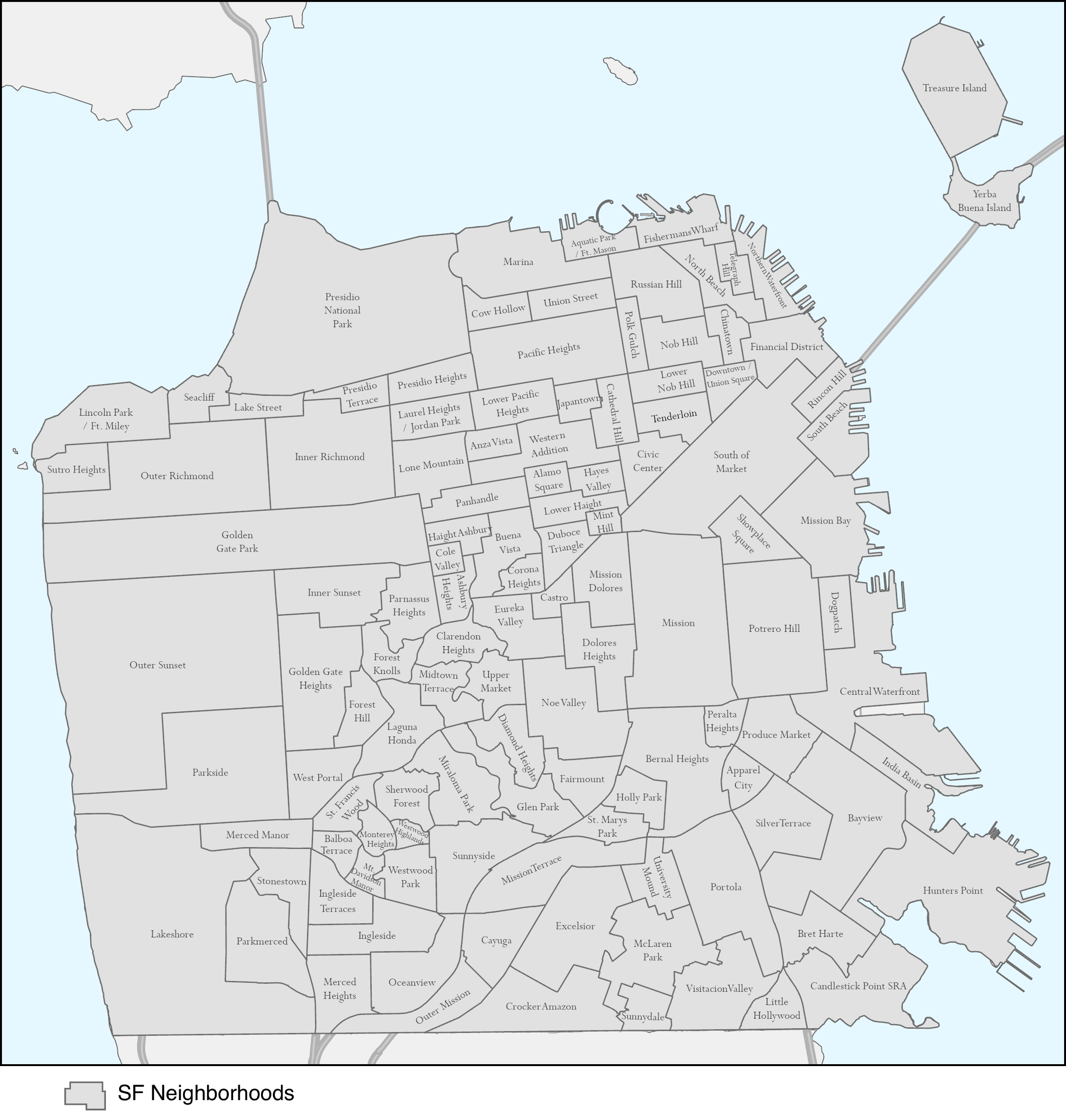

Supplement: S1 Fig — (PNG) [file pone.0327795.s001.png]
