# Supplementary material for: Privy by the Bay: Emerging hotspot analysis of 311 reports of human/animal waste near San Francisco Pit Stop locations, 2009–2022
Source: PLoS One. 2025 Aug 14;20(8):e0327795. doi: 10.1371/journal.pone.0327795 (PMC12352649; doi:10.1371/journal.pone.0327795)
Supplement: Attachment — Submitted filename: PONE-D-25-03015.docx [file pone.0327795.s002.docx]

**PONE-D-25-03015**

**Overall**

I think this is a well-conducted study. The main concern with the article is that there’s now convincing evidence that most of the fecal matter on SF sidewalks may not be from humans, but rather from canines. This needs to be more fully addressed to better identify the best policies to reduce fecal matter on sidewalks.

**Abstract**

- Consider adding public toilet or public sanitation to key words

**Introduction**

- I think it’s important to highlight that much of the fecal waste is potentially from dogs. News sources suggest high levels of dog ownership among the unhoused population: [article](https://abc7news.com/lives-of-san-franciscos-unhoused-pet-owners-and-support-they-need/14643639/). More importantly, a *Science of the Total Environment* study looked at human markers in fecal samples collected from sidewalks in SF and found that most of the fecal waste on SF sidewalks is likely to be from canines: <https://doi.org/10.1016/j.scitotenv.2024.170139>. In that study, the authors set out to collect human feces only by selecting larger fecal samples (i.e. likely to be human based on size), but only a small fraction of the samples were identified as human.

**Methods**

- Can you describe more about how public restrooms, other than Pit Stops, were identified and labeled? Fire departments vs libraries, etc.

**Results**

- Line 134. Can you add the range of reports for the 34 neighborhoods with over 1,000 reports?
- Line 131-135. There’s mention of 117 neighborhoods, but the categories discussed are 4 neighborhoods with lots of report; 34 neighborhoods with over 1,000 reports and 7 with less than 1 report per year. What about the other 72 neighborhoods?
- Lines 131-138. Can you also add the annual reports by neighborhood categories in the most recent year?

**Discussion**

- Can you discuss how certain public restrooms may affect individuals’ willingness to use them? For example, in fire departments or police departments that have public toilets, I doubt people know about them or feel comfortable using them.
- Line 205. For this sentence (“While this result seems promising, the bulk of the city failed to provide similarly optimistic results.”), I think there may need to be additional information added here. It seems important to mention that most of the reports occur in 4 neighborhoods (percent?).
- Additionally, I think it’s important to recognize that interventions for dog feces management may require novel approaches, and public toilets won’t likely solve that problem. Please add literature on what interventions might work if this is mostly canine feces.
- Lines 281-282. I think “much of the city” is a mischaracterization; I’d suggest that you give some estimate of the neighborhoods (e.g., one-third of the neighborhoods had…) in each of the categories: persistent, sporadic and intensifying.
- There are a lot of benefits of public toilets (e.g., emergency situations for Uber and Amazon drivers), which are missing in the discussion. See: “Legislative influence on the decline of public toilet provision in the UK: a case study of Leeds city” DOI: [10.2166/washdev.2024.380](https://doi-org.libproxy.berkeley.edu/10.2166/washdev.2024.380)
