# Supplementary material for: Privy by the Bay: Emerging hotspot analysis of 311 reports of human/animal waste near San Francisco Pit Stop locations, 2009–2022
Source: PLoS One. 2025 Aug 14;20(8):e0327795. doi: 10.1371/journal.pone.0327795 (PMC12352649; doi:10.1371/journal.pone.0327795)
Supplement: Attachment — Submitted filename: Comments_PONE-D-25-03015_TI.docx [file pone.0327795.s003.docx]

**Privy by the Bay: Emerging hotspot analysis of 311 reports of human/animal waste near San Francisco Pit Stop locations, 2009-2022**

Overall:

The manuscript has some potential to create a new database and identify the major spatial areas of interventions for waste management but needs streamlining. This manuscript has used an emerging tool which pave the way of application of such tool in varied way that is appreciable. I want to see a well-structured and coherent manuscript with fundamental chronology such as Introduction, Literature review/conceptual framework, Methods and Tools (Study area, tools, data, data processing, analysis process), Results, Discussion, Conclusion. With the revision, I believe, the authors can contribute more to the research community.

Criteria wise comments:

| Criteria | Comments in brief |
| --- | --- |
| The study presents the results of original research. | Partly, but the study only presented output of a tool where research questions are not explicit. |
| Results reported have not been published elsewhere. | Yes. However, authors are suggested to follow and cite Barker, T., Capone, D., Amato, H. K., Clark, R., Henderson, A., Holcomb, D. A., ... & Brown, J. (2023). Public toilets have reduced enteric pathogen hazards in San Francisco. *PLoS Water*, *2*(8), e0000152. |
| Experiments, statistics, and other analyses are performed to a high technical standard and are described in sufficient detail. | Technically sound but lacks coherence. |
| Conclusions are presented in an appropriate fashion and are supported by the data. | There is no conclusion. |
| The article is presented in an intelligible fashion and is written in standard English. | Yes. But there are some missing links between paragraphs. |
| The research meets all applicable standards for the ethics of experimentation and research integrity. | Yes. |
| The article adheres to appropriate reporting guidelines and community standards for data availability. | Yes. |

| **Section and line number** | **Comments** |
| --- | --- |
| Title |  |
| 01 | The authors can re-think about the title to be more graspable to the general reader. To me, the current title needs a reshaping for more clearance. The title shows a trend between 2009-2022 while the analysis does provide results between 2009-2015 which create confusion. |
|  |  |
| Abstract |  |
| 22-36 | The abstract should be revised as this does not convey total story. The abstract is written in a split way which needs to be written in a way so that readers can connect from start to the end. Authors can start writing by introducing the objective of the study at first followed by the key research problem. Then write 2-3 sentences to justify the need of this research in the local context. Authors can see the way of writing abstract thought the suggested papers are not in line with the topic. Still to get idea, authors are requested to follow “Gui, B., Bhardwaj, A., & Sam, L. (2024). Revealing the evolution of spatiotemporal patterns of urban expansion using mathematical modelling and emerging hotspot analysis. *Journal of Environmental Management*, *364*, 121477.” And Ghanghermeh, A., Roshan, G., Asadi, K., & Attia, S. (2024). Spatiotemporal analysis of urban heat islands and vegetation cover using emerging hotspot analysis in a humid subtropical climate. *Atmosphere*, *15*(2), 161. |
| 23 | “Access to basic sanitation facilities is a significant challenge for homeless populations”- there is no review on this claim/conceptualisation in the manuscript. Also, there is no findings why this is a significant challenge? |
| 25-26 | “The Pit Stop Program launched in 2014, introducing clean and safe public restrooms to high need areas”- this sentence is unnecessary in abstract. This sentence does not connect with the previous sentence. |
| 26 | Provide the tools used in hotspot analysis for more clarity |
| 26-27 | Introduce the study area and level of spatial analysis such as neighbourhood here. |
| 29 | “Waste reports in San Francisco showed an upward trend from 2009 to 2022”- Authors are suggested to re check whether 2009 is incorporated and placed in the analysis and findings. |
| 39 | Key word ‘sanitation’ and ‘urban health’- how is this related to the study while authors did not mention health issues/impacts in the manuscript? These two words do not seem to be keyword. |
|  |  |
| Introduction |  |
| 42-46 | I was searching for references. These claims should be cited from global to local perspective. |
| 46-47 | Redundancy with sentence line 41-42. |
| 48 | “In a 2019 report”- define 2019 report. |
| 71 | “Amato et al. found evidence”- citation is not fulfilled |
| 74-75 | “Emerging Hot Spot Analysis (EHSA) of reports of human/animal waste”- introduce reader with this method, its pros, and cons over. Authors can be benefited from- Xu, B., Qi, B., Ji, K., Liu, Z., Deng, L., & Jiang, L. (2022). Emerging hot spot analysis and the spatial–temporal trends of NDVI in the Jing River Basin of China. *Environmental Earth Sciences*, *81*(2), 55. |
| 75 | To me the study objective is not clear. The aim of the study does not reflect what research question authors are trying to answer. What is the research gap actually? |
|  | What is ‘pit stop’? |
|  | The introduction section lacks a wider literature review. Authors are suggested to provide a theoretical framework/literature review to make understand the critical aspect of the study. What is the global and regional context of the study? Literature review and conceptual framework is essential. |
|  | The introduction section, to me, is not coherent. Authors are suggested to revisit it properly. |
|  |  |
| Methods |  |
| 83 | “Duplicates were identified using Amato et al.’s methodology”- cite properly |
| 84 | “any records with “dup”- define ‘dup’. Please ensure you provide detail about any ambiguous words well ahead for the readers who does not know about it. |
| 86-97 | Authors can think of a data table from this paragraph for more readability. |
| 88 | “partitioned by season and neighborhood”- I did not find any analysis showing seasonal variations in hot-spot. The result section also does not reflect any output regarding this except a figure which does not convey what critical arguments are coming?. Authors are essentially need to connect the methods and result section. |
|  | The authors did not provide sufficient data to prove the effects of seasonality and their impacts on neighbourhoods/county. |
| 91-97 | These texts need more clarification. This is confusing what authors are actually trying to convey? |
|  | “waste report density were calculated for each neighborhood”- clarify the process of waste report density calculation. |
| 98-100 | Authors indicated two temporal and spatial dataset here (OSM data which is latest, and The pit stop data). How did authors link two separate databases? How did the authors managed to link two temporal data? Is there any anomaly? |
| 103 | “to examine spatiotemporal clustering trends”- no analysis included in the study about the spatiotemporal clustering trends. Please provide details. |
| 105 | “space-time netCDF cube data structures”- provide details on it. |
| 107-108 | “approximate comfortable walking distance”- what is the distance and what is the standard? Provide citation |
| 109 | “FDR correction”- what is FDR? Full meaning? Please ensure you provide full meaning of each abbreviation throughout the texts. |
| 113 | “emerging hot spot categories were evaluated”- for what this evaluation is carried out? |
| 115 | “A weighted Pit Stop Accessibility Score was calculated”- please provide the process of weighing. |
| 115-116 | “to evaluate if the intensity of the Pit Stop intervention,”- authors at result section did not provide any results or mentioned any process in the method section about the calculation of intensity of Pit Stop interventions. Please provide details. |
| 117 | “Every walking distance hexagon”- how did authors created hexagon? What is the measurement of walking distance? |
|  | Authors can benefit from the paper about how to write an effective method from “Morckel, V., & Durst, N. (2023). Using Emerging Hot Spot Analysis to explore spatiotemporal patterns of housing vacancy in Ohio metropolitan statistical areas. *Urban Affairs Review*, *59*(1), 309-328.”  Mondal, S., Singh, D., & Kumar, R. (2022). Crime hotspot detection using statistical and geospatial methods: a case study of Pune City, Maharashtra, India. *GeoJournal*, *87*(6), 5287-5303. |
| 117-119 | Please provide details how did the authors come up with the scoring? |
| 120 | “weighted by the appropriate number of years”- provide details about the weighing procedure. |
|  | Why the EHSA is suitable for your study? Is there any spatial autocorrelation of the variables? |
|  | Method should have sub-section such as study area selection, variables to analyse, tools used. |
|  | A flow chart of methodology can be produced. |
|  | A study area map is essential to locate where the study area is located from global/regional perspective, which is missing now. |
|  | A table consisting variables, tools used can be beneficial for the readers. |
|  |  |
| Results |  |
| 131 | “There was also substantial spatial variation in waste reports.”- describe the reasons. |
| 139 | “spread across 13 neighborhoods”- how did the authors define neighbourhoods? How many neighbourhoods are there in total? Why did the authors create hexagon while the analysis unit is neighbourhood? Did the authors sample these 13 neighbourhoods? If yes, then why? |
|  | What is the impact of creating Hexagon in the analysis except demarcating walking distance? How the authors conceptualised walking distance? How many walking distance hexagons are created? Any standard? Please provide reference and details. |
| 139-153 | These texts need re-writing for greater readability specially from line 141. |
| 151 | Figure 2: annual trend should have starting year 2009 as title referred. |
| 157-159 | “Pit Stops tend to be concentrated in areas with the highest count of waste reports, most notably in the Tenderloin neighborhood.”- Is this concentration statistically significant? Is there any impact of neighbourhood character/spatial adjacency in the analysis? Provide the details on factors affecting the concentration in particular neighbourhood. |
| 161 | “EHSA results for seasonal datasets were similar and are not reported.”- if it is so, then clarify this in the method section. Authors may drop this part from the manuscript (method) as there is no effect of seasons in the analysis. |
| 168-170 | Is this a problem of the tool? Please describe. |
| 171 | “Hot spots were detected primarily in the north-eastern quadrant of the city”- how did the authors classified the quadrant? Method section did not focus on this process. |
| 172 | “Substantial portions of the city”- what is substantial? Please provide data for clarity such that ‘*65% of the city’.* |
| 178 | Table 1 column 1 align the text as ‘top left’. How did the authors classified the ‘'trend categories’?. What are the meaning of the categories (not asking about the definition but the actual findings based on the categories)? |
|  | How did authors calculated the ‘average pit stop accessibility score’? needs to be detailed. |
| 192 | “average score of 0 for new hot spots”- is there any particular reason for 0 average? If yes then clarify. |
|  | Maps should be detailed out. Figure 3 does not tell for which year it is prepared. What about the temporal changes? It is essential to produce temporal maps to see whether there are significant changes in spatial pattern of the subject matter. |
|  | The result is presented well. However, critical arguments are missing and essential to produce. |
|  | There is an inconsistency in presentation of results. The PI Stop programme is likely to had a start in 2014 but the authors used data from 2009. How did the authors linked the temporal dataset? |
|  | What are the arguments the analysis creating? Identify the main findings in bullet points at the end of the result section. Write the result section chronologically based on the aim/theme of the study. |
|  |  |
| Discussion |  |
| 224-232 | This full paragraph is not suitable here. This is about the study area may be. Suggesting to transfer it to Intro or Method (study area sub section). Also, the paragraph does not have any importance with the study. |
| 234 | “this population is perhaps most affected”- how did authors claim this statement? What are the factors of ‘affected’? |
|  |  |
|  | The discussion section seriously lacks justification of arguments in comparison to the earlier studies. Authors are requested to revisit the discussion section. Authors can see “Mondal, S., Singh, D., & Kumar, R. (2022). Crime hotspot detection using statistical and geospatial methods: a case study of Pune City, Maharashtra, India. *GeoJournal*, *87*(6), 5287-5303.” |
|  | Start the discussion section by re-uttering the study objectives and how did the authors fulfil the objective(s). Then authors can discuss the main claims on the results. Re-write the section. |
|  |  |
| Limitations |  |
| 282 | “much of the city exhibited persistent, sporadic, or intensifying hotspot trends.”- I did not find any trend analysis or any map. Spatial unit of the study is neighbourhood. From where the unit ‘city’ come? |
|  |  |
| Conclusion* |  |
|  | This is astonishing that the manuscript does not have ‘conclusion’. Authors are requested to include a conclusion section with specific policy implications and way forward. This is crucial. |
|  | The study cited most of the google findings rather than actual scholarly articles. Please include scholarly articles where appropriate to theorize the idea and connect to the findings. |
|  | Only doing a hot spot analysis does not seem to be the primary objective here Authors need to bring arguments in line with urban health, sanitation practices, well-being, neighbourhood characteristics etc where possible. How will the result affect the decision-making process in USA? |
