# Supplementary material for: Privy by the Bay: Emerging hotspot analysis of 311 reports of human/animal waste near San Francisco Pit Stop locations, 2009–2022
Source: PLoS One. 2025 Aug 14;20(8):e0327795. doi: 10.1371/journal.pone.0327795 (PMC12352649; doi:10.1371/journal.pone.0327795)
Supplement: Attachment — Submitted filename: Response to Reviewers.docx [file pone.0327795.s004.docx]

We are incredibly grateful for the thoughtful review of our manuscript and feedback provided by all of the reviewers. Below, please find responses to all comments made (• bulleted, in blue).

5. Review Comments to the Author

Please use the space provided to explain your answers to the questions above. You may also include additional comments for the author, including concerns about dual publication, research ethics, or publication ethics. (Please upload your review as an attachment if it exceeds 20,000 characters)

Reviewer #1: Please see attached comments.


Overall
I think this is a well-conducted study. The main concern with the article is that there’s now convincing evidence that most of the fecal matter on SF sidewalks may not be from humans, but rather from canines. This needs to be more fully addressed to better identify the best policies to reduce fecal matter on sidewalks.

- We are grateful to this reviewer’s careful edits and suggestions to improve our manuscript. We believe we have included an overview of the recent evidence this reviewer has brought to our attention, and incorporated a summation of this work in our revision in both the Discussion and Limitations sections of the manuscript as a possible explanation for our results and an opportunity for future work to distinguish between human and animal waste to more accurately estimate the impact of interventions like the Pit Stop program.

Abstract
Consider adding public toilet or public sanitation to key words

- We have added “public sanitation” as a key word. We appreciate the suggestion.

Introduction
I think it’s important to highlight that much of the fecal waste is potentially from dogs. News sources suggest high levels of dog ownership among the unhoused population: article. More importantly, a Science of the Total Environment study looked at human markers in fecal samples collected from sidewalks in SF and found that most of the fecal waste on SF sidewalks is likely to be from canines: [https://doi.org/10.1016/j.scitotenv.2024.170139](https://urldefense.com/v3/__https:/doi.org/10.1016/j.scitotenv.2024.170139__;!!LFqOYw!r35FnLR65-KcxRX_MakmVczbN_-E-27F3EK74_pWu2UQz_5lz2tyCC3J3sQUyRQ7AHz2a3ulNTDVOuYQbA$). In that study, the authors set out to collect human feces only by selecting larger fecal samples (i.e. likely to be human based on size), but only a small fraction of the samples were identified as human.

- We are grateful for this comment, and for drawing our attention to this recent evidence that is highly relevant to our work. We have added a section to both the Discussion and Limitations section of the manuscript to highlight how our study cannot distinguish between 311 reports of human and animal (likely canine, as was found in the referenced and cited paper) waste. We believe this improves the paper, and provides a possible explanation to the results we observed in our study (i.e., the limited impact of the presence of Pit Stops may be due to reports disproportionately reporting canine rather than human waste).

Methods
Can you describe more about how public restrooms, other than Pit Stops, were identified and labeled? Fire departments vs libraries, etc.

- We are happy to provide additional information and clarification regarding the identification of other public restrooms. As such, we have supplemented our Methods section to clarify that facilities with the “toilet” tag were included, and that toilets accessible only to customers (toilets:access=customers) were not included. We also provided supplemental information to the Limitations section, where we had previously described the data quality and completeness of the OSM public restroom layer as a limitation of the study. We have expanded this limitation with a more detailed description of OSM data and how they are likely not comprehensive or completely accurate at a given time.

Results
Line 134. Can you add the range of reports for the 34 neighborhoods with over 1,000 reports?

- We appreciate the opportunity to provide additional information and clarification here. We have made the following edits to the sentence at (former) Line 134: “For example, the four neighborhoods with the most total reports (3.4% of all neighborhoods) each had over 10,000 reports (Tenderloin, n = 39,558; South of Market, n = 39,154; Mission, n = 31,610; and Lower Nob Hill, n = 11,180), 30 additional neighborhoods (25.6% of all neighborhoods) had 1,000 or more reports (ranging from n = 1,008 in Excelsior to n = 8,536 in Civic Center), and 83 neighborhoods (70.9%) had fewer than 1,000 reports during the study time period (ranging from n = 1 in Yerba Buena Island to n = 922 in Inner Sunset).”

Line 131-135. There’s mention of 117 neighborhoods, but the categories discussed are 4 neighborhoods with lots of report; 34 neighborhoods with over 1,000 reports and 7 with less than 1 report per year. What about the other 72 neighborhoods?

- We are grateful for the opportunity to clarify this information, as it was not worded clearly in our initial draft. We have made the following edits to the sentence at (former) Lines 131-135 to clarify where all of the neighborhood fell into 3 bins (10,000+, 1,000-9,999, and less than 1,000): “For example, the four neighborhoods with the most total reports (3.4% of all neighborhoods) each had over 10,000 reports (Tenderloin, n = 39,558; South of Market, n = 39,154; Mission, n = 31,610; and Lower Nob Hill, n = 11,180), 30 additional neighborhoods (25.6% of all neighborhoods) had 1,000 or more reports (ranging from n = 1,008 in Excelsior to n = 8,536 in Civic Center), and 83 neighborhoods (70.9%) had fewer than 1,000 reports during the study time period (ranging from n = 1 in Yerba Buena Island to n = 922 in Inner Sunset).”

Lines 131-138. Can you also add the annual reports by neighborhood categories in the most recent year?

- Thank you for the comment. We have added a sentence to incorporate this feedback, describing broadly how reports were distributed among the 117 neighborhoods in just the most recent year of data: “In just the most recent year of data (2022), the same four neighborhoods each had over 3,500 reports, 42 neighborhoods (35.9% of all neighborhoods) had more than 100 reports, and the remaining 71 (60.7%) had fewer than 100 reports.”

Discussion
Can you discuss how certain public restrooms may affect individuals’ willingness to use them? For example, in fire departments or police departments that have public toilets, I doubt people know about them or feel comfortable using them.

- We appreciate and agree with this comment. We have added a paragraph to the Discussion section to address the issue of stigma and accessibility of public restrooms to people experiencing homelessness, and call into question how accessible these non-Pit Stop locations may be for individuals experiencing homelessness. We have also expanded on the limitations of OSM data to identify public restrooms in the Limitations section to incorporate this point.

Line 205. For this sentence (“While this result seems promising, the bulk of the city failed to provide similarly optimistic results.”), I think there may need to be additional information added here. It seems important to mention that most of the reports occur in 4 neighborhoods (percent?).

- We appreciate the opportunity to provide additional context for our results here. We have edited this sentence in the following way, based on this feedback: “While this result seems promising, the bulk of the city failed to provide similarly optimistic results. The majority of waste reports (53.5%) were reported from four neighborhoods (3.4% of all neighborhoods), all with over 10,000 reports. Although the Tenderloin neighborhood demonstrated a large area of diminishing hotspots, the other three neighborhoods with over 10,000 reports (South of Market, Mission, and Lower Nob Hill) showed few or no areas of diminishing hotspots. Additionally, surrounding the group of diminishing hot spot hexagons concentrated in the Tenderloin neighborhood are consecutive and intensifying hot spots.”

Additionally, I think it’s important to recognize that interventions for dog feces management may require novel approaches, and public toilets won’t likely solve that problem. Please add literature on what interventions might work if this is mostly canine feces.

- We are grateful for this comment, and for the opportunity to draw attention to other research into canine waste reduction efforts, which may be needed in parallel to interventions for public toilets. We have expanded the paragraph in our Discussion section that describes how canine feces may be well-represented in our data set, and have added information on pet waste reduction campaigns that have been successful in other areas of the country.

Lines 281-282. I think “much of the city” is a mischaracterization; I’d suggest that you give some estimate of the neighborhoods (e.g., one-third of the neighborhoods had…) in each of the categories: persistent, sporadic and intensifying.

- We appreciate this comment, and recognize the non-specificity of the original word choice. We have edited this sentence to read: “Overall, reports of waste to San Francisco 311 increased from 2009 to 2022, and 5.7% (n = 326) of the walking-distance hexagons across 37 of the city’s 117 neighborhoods (32%) exhibited new, consecutive, persistent, sporadic, or intensifying hotspot trends.”

There are a lot of benefits of public toilets (e.g., emergency situations for Uber and Amazon drivers), which are missing in the discussion. See: “Legislative influence on the decline of public toilet provision in the UK: a case study of Leeds city” DOI: 10.2166/washdev.2024.380

- We are grateful for this comment and the opportunity to integrate this background in support of the need for more public toilets for multiple populations, not just people experiencing homelessness. We have added this note to our Discussion section, as well as the provided reference and several others supporting the point.

Reviewer #2: Thank your for your excellent work. The manuscript has some potential to create a new database and identify the major spatial areas of interventions for waste management but needs streamlining. This manuscript has used an emerging tool which pave the way of application of such tool in varied way that is appreciable. I want to see a well-structured and coherent manuscript with fundamental chronology such as Introduction, Literature review/conceptual framework, Methods and Tools (Study area, tools, data, data processing, analysis process), Results, Discussion, Conclusion. With the revision, I believe, the authors can contribute more to the research community. Detail comments are attached.

- We appreciate this suggestion to improve the readability and organization of our manuscript. We have added subheadings in addition to the main headings, based on this comment and the included suggestions.

Overall:

The manuscript has some potential to create a new database and identify the major spatial areas of interventions for waste management but needs streamlining. This manuscript has used an emerging tool which pave the way of application of such tool in varied way that is appreciable. I want to see a well-structured and coherent manuscript with fundamental chronology such as Introduction, Literature review/conceptual framework, Methods and Tools (Study area, tools, data, data processing, analysis process), Results, Discussion, Conclusion. With the revision, I believe, the authors can contribute more to the research community.

Criteria wise comments:

| Criteria | Comments in brief |
| --- | --- |
| The study presents the results of original research. | Partly, but the study only presented output of a tool where research questions are not explicit.   - We are grateful for this feedback. We have added study aims just before the Methods section to incorporate this suggestion and make our research aims more explicit and clear to readers. |
| Results reported have not been published elsewhere. | Yes. However, authors are suggested to follow and cite Barker, T., Capone, D., Amato, H. K., Clark, R., Henderson, A., Holcomb, D. A., ... & Brown, J. (2023). Public toilets have reduced enteric pathogen hazards in San Francisco. *PLoS Water*, *2*(8), e0000152.   - We are grateful to this reviewer for bringing this article to our attention. We have added a discussion of this and another related article to our Discussion section in addressing comments made by Reviewer 1 to highlight the prevalence of canine feces as a public health issue in San Francisco. |
| Experiments, statistics, and other analyses are performed to a high technical standard and are described in sufficient detail. | Technically sound but lacks coherence.   - We are grateful for this feedback. We have added sub-headings to distinguish results as part of Aim 1 or Aim 2 in an effort to increase clarity and coherence of the results. |
| Conclusions are presented in an appropriate fashion and are supported by the data. | There is no conclusion.   - We appreciate the opportunity to add a Conclusion section to our manuscript. We have updated the paper so that the final paragraph is now a conclusion to address this comment. |
| The article is presented in an intelligible fashion and is written in standard English. | Yes. But there are some missing links between paragraphs.   - We have heavily edited our manuscript based on the comments made by this and other reviewers. We believe our manuscript is more easily understood and flows much better thanks to them. |
| The research meets all applicable standards for the ethics of experimentation and research integrity. | Yes. |
| The article adheres to appropriate reporting guidelines and community standards for data availability. | Yes. |

| **Section and line number** | **Comments** |
| --- | --- |
| Title |  |
| 01 | The authors can re-think about the title to be more graspable to the general reader. To me, the current title needs a reshaping for more clearance. The title shows a trend between 2009-2022 while the analysis does provide results between 2009-2015 which create confusion.   - We appreciate this feedback. Our analysis does span 2009-2022, so we would like to eliminate the confusion about the time frame of the data we used. We have re-read our manuscript and edited a line in our Methods to clarify that half of the Tenderloin’s Pit Stop locations were installed between 2014-2015, and not that our analyses stopped at this point. We would be happy to provide additional clarity, if needed. |
|  |  |
| Abstract |  |
| 22-36 | The abstract should be revised as this does not convey total story. The abstract is written in a split way which needs to be written in a way so that readers can connect from start to the end. Authors can start writing by introducing the objective of the study at first followed by the key research problem. Then write 2-3 sentences to justify the need of this research in the local context. Authors can see the way of writing abstract thought the suggested papers are not in line with the topic. Still to get idea, authors are requested to follow “Gui, B., Bhardwaj, A., & Sam, L. (2024). Revealing the evolution of spatiotemporal patterns of urban expansion using mathematical modelling and emerging hotspot analysis. *Journal of Environmental Management*, *364*, 121477.” And Ghanghermeh, A., Roshan, G., Asadi, K., & Attia, S. (2024). Spatiotemporal analysis of urban heat islands and vegetation cover using emerging hotspot analysis in a humid subtropical climate. *Atmosphere*, *15*(2), 161.   - We are grateful for this feedback and the opportunity to revise. We have referred to the articles mentioned, and in our abstract we have now added a connection to the objective of the study to sentence 1, which is now followed by 2 sentences of research/background of the local context before describing the methods and results. |
| 23 | “Access to basic sanitation facilities is a significant challenge for homeless populations”- there is no review on this claim/conceptualisation in the manuscript. Also, there is no findings why this is a significant challenge?   - We appreciate this feedback and the opportunity to provide a greater breadth of evidence to support our claim. This sentence appears in the abstract, and so we cannot provide references. However, we have added several additional citations to support the significance of the challenge in the first sentence of the Introduction to help ground the need for public health interventions and address this comment. |
| 25-26 | “The Pit Stop Program launched in 2014, introducing clean and safe public restrooms to high need areas”- this sentence is unnecessary in abstract. This sentence does not connect with the previous sentence.   - We are grateful for this feedback, and the opportunity to increase the readability of our abstract. We have added a transitional clause to connect the main ideas of these two sentences, so they now read: “San Francisco, California, has a large population of people experiencing homelessness and limited public restrooms. To increase access to public restrooms among this population in the city, the Pit Stop Program launched in 2014, introducing clean and safe public restrooms to high-need areas.” |
| 26 | Provide the tools used in hotspot analysis for more clarity   - We have added the software to this sentence to increase clarity. |
| 26-27 | Introduce the study area and level of spatial analysis such as neighbourhood here.   - We have added the study area and the neighborhood level of analysis here, as recommended by this reviewer. |
| 29 | “Waste reports in San Francisco showed an upward trend from 2009 to 2022”- Authors are suggested to re check whether 2009 is incorporated and placed in the analysis and findings.   - The data include reports beginning on January 1, 2009, as stated in the second paragraph of the Data Sources and Processing section: “The overall data were restricted to reports made in complete years of data (between January 1, 2009 and December 31, 2022; n = 230,270).” Figures 1 and 2 do not label every year for fear of overcrowded label axes, but data are reported in both going back to 2009. If there is a specific location in the manuscript, tables, or figures where it seems like 2009 is not included in the data, please do let us know so we can correct it! |
| 39 | Key word ‘sanitation’ and ‘urban health’- how is this related to the study while authors did not mention health issues/impacts in the manuscript? These two words do not seem to be keyword.   - We are grateful for this feedback. We have removed “urban health” as a key word. Based on the feedback of another reviewer, we have modified “sanitation” to be “public sanitation” at their recommendation. |
|  |  |
| Introduction |  |
| 42-46 | I was searching for references. These claims should be cited from global to local perspective.   - We appreciate this comment to expand the background here. We have added additional references and cited them in the order recommended. |
| 46-47 | Redundancy with sentence line 41-42.   - We have removed this sentence. |
| 48 | “In a 2019 report”- define 2019 report.   - We have clarified that this is a report authored by the Pacific Institute. |
| 71 | “Amato et al. found evidence”- citation is not fulfilled   - We have corrected this by completing the citation. |
| 74-75 | “Emerging Hot Spot Analysis (EHSA) of reports of human/animal waste”- introduce reader with this method, its pros, and cons over. Authors can be benefited from- Xu, B., Qi, B., Ji, K., Liu, Z., Deng, L., & Jiang, L. (2022). Emerging hot spot analysis and the spatial–temporal trends of NDVI in the Jing River Basin of China. *Environmental Earth Sciences*, *81*(2), 55.   - We appreciate this attention to the methodological context. We have added this citation and brief description of the advantage of the analysis. |
| 75 | To me the study objective is not clear. The aim of the study does not reflect what research question authors are trying to answer. What is the research gap actually?   - We appreciate the attention to this oversight and the opportunity to provide additional clarity. We have expanded the final paragraph of the introduction to include more concrete research aims and have used those aims as subheadings in the Results section to help structure and organize the manuscript. |
|  | What is ‘pit stop’?   - We have clarified that the Pit Stop Program is the creation and maintenance of public restrooms for people who do not have consistent access to them (in the Introduction section). |
|  | The introduction section lacks a wider literature review. Authors are suggested to provide a theoretical framework/literature review to make understand the critical aspect of the study. What is the global and regional context of the study? Literature review and conceptual framework is essential.   - We appreciate this feedback and the opportunity to provide a more comprehensive overview of the research to date. As such, we have added additional literature in the Introduction section. |
|  | The introduction section, to me, is not coherent. Authors are suggested to revisit it properly.   - We are grateful for this feedback, and the opportunity to provide more clarity for readers. We have edited the Introduction section, including rearranging paragraphs to improve flow, with special attention paid to transitions to ensure that one idea follows logically from the prior one. |
|  |  |
| Methods |  |
| 83 | “Duplicates were identified using Amato et al.’s methodology”- cite properly   - We have added the complete citation here. |
| 84 | “any records with “dup”- define ‘dup’. Please ensure you provide detail about any ambiguous words well ahead for the readers who does not know about it.   - We appreciate the opportunity to provide this clarification. We have added additional language to make this step clearer to all readers: “…excluding any records with “dup” (short for “duplication” and related words to remove duplicated reports as identified by the Responsible Agency)…” |
| 86-97 | Authors can think of a data table from this paragraph for more readability.   - We appreciate this comment, and the opportunity to provide a more readable section with the inclusion of a table. We have moved most of the language from this paragraph to a new Table 1 that describes inclusion/exclusion criteria, and n values at each step. |
| 88 | “partitioned by season and neighborhood”- I did not find any analysis showing seasonal variations in hot-spot. The result section also does not reflect any output regarding this except a figure which does not convey what critical arguments are coming?. Authors are essentially need to connect the methods and result section.   - We are grateful for this comment and the opportunity provide additional clarity. As stated in the results section, the EHSA results for the seasonal datasets were very similar to the overall EHSA results and were thus not reported, as there would be 4 figures or 1 figure with 4 panels that would look virtually identical to panel B in Figure 3. Although we anticipated effects of seasonality, because we did not observe strong EHSA effects of seasonality, we tried to streamline the reporting of our results by not reporting analyses that ultimately were not interesting. Although there are temporal trends that can be observed in Figure 1, no consistent pattern of one season dominating the volume of reports over others is evident. We have added a sentence to the Methods section to let readers know that the data did not support effects of seasonality, and will not be reported. |
|  | The authors did not provide sufficient data to prove the effects of seasonality and their impacts on neighbourhoods/county.   - We appreciate this feedback, and the opportunity to provide better guidance for readers to anticipate and contextualize our results. As stated above, although we allowed for the possibility of effects of seasonality, no effects of seasonality were observed. We feared removing the descriptive analysis by seasonality (Figure 1) would make readers think we had not considered seasonality, so we retained that information to demonstrate that although temporal trends were observed, they were not driven by seasonal effects. We do not discuss effects of seasons in the Discussion section for this reason. We have now added a sentence to the Methods section to let readers know that the data did not support effects of seasonality, and will not be reported. |
| 91-97 | These texts need more clarification. This is confusing what authors are actually trying to convey?   - We appreciate the opportunity to provide clarity for readers here. We have edited this section to move most of the text into a new Table 1, based on this reviewer’s helpful suggestion. We hope that these edits have clarified the text highlighted here. |
|  | “waste report density were calculated for each neighborhood”- clarify the process of waste report density calculation.   - We appreciate this suggestion, and have clarified that density was calculated in thousands of reports per square kilometer for each neighborhood. |
| 98-100 | Authors indicated two temporal and spatial dataset here (OSM data which is latest, and The pit stop data). How did authors link two separate databases? How did the authors managed to link two temporal data? Is there any anomaly?   - We are grateful for this feedback to provide additional clarity. Both data sets were converted to a point layer so they could be spatially represented in the same analyses. Linking is thus done based on latitude and longitude, which were inherent in the OSM data, and identified based on known addresses for the Pit Stop data. Temporal information is known about the Pit Stop locations (i.e., when these locations were opened), and is provided in Figure 2. However, temporal information for public restrooms is unknown, and is a known limitation of our study, which we have described in our Limitations section. To provide additional clarity, we have added the following sentence to our Data Analysis section: “A single point layer representing public restrooms (including Pit Stops) was created for subsequent analyses.” |
| 103 | “to examine spatiotemporal clustering trends”- no analysis included in the study about the spatiotemporal clustering trends. Please provide details.   - We appreciate this feedback. We are trying here to describe EHSA, which examines trends in incidence over time in pre-defined clusters. However, we recognize that the use of the word “cluster” may be confusing, and thus we have removed it here. |
| 105 | “space-time netCDF cube data structures”- provide details on it.   - We appreciate this comment and the opportunity to provide more information for readers. We have added more information about space-time netCDF cubes, as well as a citation from different literature that nevertheless has a good explanation of the advantages of analyzing spatiotemporal information in this way. |
| 107-108 | “approximate comfortable walking distance”- what is the distance and what is the standard? Provide citation   - We are grateful for this comment and the opportunity to provide readers with more context regarding this analytical decision. We have added the following sentences with citations to our Data Analysis section where an appropriate walking distance is first described: “No universal standard for a comfortable or appropriate walking distance to restrooms exists, although multiple recommendations have been suggested by different organizational bodies around the world for different situations and stakeholders, including 60 meters or ~0.9 hectares (39), 100 meters or ~2.6 hectares (40), and 200 feet or ~0.9 hectares (41). Given the lack of consensus, we selected an intermediate distance of 1.8 hectares.” |
| 109 | “FDR correction”- what is FDR? Full meaning? Please ensure you provide full meaning of each abbreviation throughout the texts.   - We have spelled out “False Discovery Rate (FDR) correction” to clarify the meaning for readers. |
| 113 | “emerging hot spot categories were evaluated”- for what this evaluation is carried out?   - We are grateful for the chance to clarify this point. We have replace the verb “evaluated” with “identified”, as this was a descriptive analysis. |
| 115 | “A weighted Pit Stop Accessibility Score was calculated”- please provide the process of weighing.   - We appreciate the opportunity to provide clarity in the calculation of this score. We have added a transition to make it clearer to readers that the following sentences describe the calculation process. |
| 115-116 | “to evaluate if the intensity of the Pit Stop intervention,”- authors at result section did not provide any results or mentioned any process in the method section about the calculation of intensity of Pit Stop interventions. Please provide details.   - As described above, we have added a transition and made several edits to make it clearer to readers that the following sentences describe the calculation process. |
| 117 | “Every walking distance hexagon”- how did authors created hexagon? What is the measurement of walking distance?   - We appreciate the opportunity to provide our readers with more information regarding this process. As described above, we have added the following sentences with citations to our Data Analysis section where an appropriate walking distance is first described: “No universal standard for a comfortable or appropriate walking distance to restrooms exists, although multiple recommendations have been suggested by different organizational bodies around the world for different situations and stakeholders, including 60 meters or ~0.9 hectares (39), 100 meters or ~2.6 hectares (40), and 200 feet or ~0.9 hectares (41). Given the lack of consensus, we selected an intermediate distance of 1.8 hectares.” |
|  | Authors can benefit from the paper about how to write an effective method from “Morckel, V., & Durst, N. (2023). Using Emerging Hot Spot Analysis to explore spatiotemporal patterns of housing vacancy in Ohio metropolitan statistical areas. *Urban Affairs Review*, *59*(1), 309-328.”  Mondal, S., Singh, D., & Kumar, R. (2022). Crime hotspot detection using statistical and geospatial methods: a case study of Pune City, Maharashtra, India. *GeoJournal*, *87*(6), 5287-5303.   - We are grateful for this feedback. We have reviewed these citations and made several edits to our Methods section. We believe this section is now much clearer to readers. |
| 117-119 | Please provide details how did the authors come up with the scoring?   - We appreciate this comment and the opportunity to provide additional clarity. As described in a comment above, we have added a transition and made several edits to make the calculation process clearer to readers. |
| 120 | “weighted by the appropriate number of years”- provide details about the weighing procedure.   - We are grateful for this feedback and the opportunity to make this section clearer to readers. As described in a comment above, we have added a transition and made several edits to make the calculation process clearer to readers. |
|  | Why the EHSA is suitable for your study? Is there any spatial autocorrelation of the variables?   - We appreciate this feedback. In response to this and other reviewer comments, we have provided additional detail about the EHSA analysis in the Data Analysis section, including how spatial autocorrelation is utilized in the discovery of hotspots. |
|  | Method should have sub-section such as study area selection, variables to analyse, tools used.   - We appreciate the opportunity to provide additional structure and organization. We have included subheadings in the Methods section to facilitate organization and readability. |
|  | A flow chart of methodology can be produced.   - We appreciate this feedback, and in response, we have included a new table to describe our methodology (now Table 2). |
|  | A study area map is essential to locate where the study area is located from global/regional perspective, which is missing now.   - We are grateful for this comment and the opportunity to provide additional context for readers outside of the United States. We have modified Figure 4 to include an inset globe pointing to San Francisco to provide that clarification from a global perspective. |
|  | A table consisting variables, tools used can be beneficial for the readers.   - We are grateful for this feedback, and the opportunity to provide additional clarity. We have added a new table listing our data sources and the relevant variables used (now Table 1). |
|  |  |
| Results |  |
| 131 | “There was also substantial spatial variation in waste reports.”- describe the reasons.   - We appreciate this feedback, and we have provided a more comprehensive overview of waste reports in all neighborhoods across the city in this paragraph. |
| 139 | “spread across 13 neighborhoods”- how did the authors define neighbourhoods? How many neighbourhoods are there in total? Why did the authors create hexagon while the analysis unit is neighbourhood? Did the authors sample these 13 neighbourhoods? If yes, then why?   - We are grateful for this feedback and the opportunity to provide clarification to readers. We have edited this sentence to provide more detail, particularly for international readers: “GIS data on all 117 San Francisco neighborhoods were downloaded from DataSF (with boundaries defined by the Mayor’s Office of Neighborhood Services in 2006)” |
|  | What is the impact of creating Hexagon in the analysis except demarcating walking distance? How the authors conceptualised walking distance? How many walking distance hexagons are created? Any standard? Please provide reference and details.   - We appreciate this feedback and the opportunity to supplement our manuscript with additional information about this process. As described above, we have added the following sentences with citations to our Data Analysis section where an appropriate walking distance is first described: “No universal standard for a comfortable or appropriate walking distance to restrooms exists, although multiple recommendations have been suggested by different organizational bodies around the world for different situations and stakeholders, including 60 meters or ~0.9 hectares (39), 100 meters or ~2.6 hectares (40), and 200 feet or ~0.9 hectares (41). Given the lack of consensus, we selected an intermediate distance of 1.8 hectares.” |
| 139-153 | These texts need re-writing for greater readability specially from line 141.   - We are grateful for this feedback, and we have revised this paragraph to increase readability. |
| 151 | Figure 2: annual trend should have starting year 2009 as title referred.   - We agree wholeheartedly with this recommendation, and we have edited the Figure 2 caption to reflect this feedback. |
| 157-159 | “Pit Stops tend to be concentrated in areas with the highest count of waste reports, most notably in the Tenderloin neighborhood.”- Is this concentration statistically significant? Is there any impact of neighbourhood character/spatial adjacency in the analysis? Provide the details on factors affecting the concentration in particular neighbourhood.   - We appreciate this feedback and the opportunity provide further clarification. The current study was a primarily descriptive analysis when it comes to trends in waste reports and characterization of those trends in San Francisco neighborhoods because of the difficulty comparing neighborhoods of different sizes. The statistical tests in our study were performed using the EHSA hexagons. Although the EHSA hexagons facilitate significance testing and analysis through the creation and comparison of standardized areas for comparison over time, they are not meaningful to stakeholders. To facilitate the usefulness of our findings for policymakers, residents, and other stakeholders, we used neighborhoods to characterize and help ground our findings with an eye toward policy changes. |
| 161 | “EHSA results for seasonal datasets were similar and are not reported.”- if it is so, then clarify this in the method section. Authors may drop this part from the manuscript (method) as there is no effect of seasons in the analysis.   - We appreciate this comment, and the assistance in helping us streamline our manuscript. We have removed this sentence and added the following sentence to the Methods section, as advised: “However, no effects of seasonality were observed in the EHSA analyses, and are therefore not reported.” |
| 168-170 | Is this a problem of the tool? Please describe.   - We are grateful for the opportunity to provide further clarification to readers. We have added information to this sentence to clarify that “no pattern detected” means no changes in space/time for human/animal waste reports: “Most of the city (93.8% of all walking-distance hexagons) was classified as “no pattern detected” meaning either the spatial clustering tests or the temporal trend tests failed to achieve statistical significance for that location, suggesting no change in human/animal waste reporting for these areas over time.” |
| 171 | “Hot spots were detected primarily in the north-eastern quadrant of the city”- how did the authors classified the quadrant? Method section did not focus on this process.   - We appreciate this feedback and the opportunity clarify. We used the term “north-eastern” to describe the general area of the city. We have clarified this by removing the word “quadrant.” |
| 172 | “Substantial portions of the city”- what is substantial? Please provide data for clarity such that ‘*65% of the city’.*   - We agree and appreciate this comment, as this language was unclear. We have revised this sentence to incorporate this feedback: “Overall, 6.2% of the city were identified as hot spots of some type, including d sporadic (1%), consecutive (3.5%), and intensifying (0.8%) hot spots, with some more limited cases of new (0.2%) and persistent (0.2%) hot spots (see Table 3 for hot spot category definitions).” |
| 178 | Table 1 column 1 align the text as ‘top left’. How did the authors classified the ‘'trend categories’?. What are the meaning of the categories (not asking about the definition but the actual findings based on the categories)?   - We are grateful for this feedback. This table has changed based on feedback from another reviewer, and thus the column 1 header is now removed. We have added the clarification that these categories are definitions provided by the EHSA analysis documentation within ArcGIS Pro, but this information has been moved up to 2.2. Data Analysis to align with feedback from another reviewer. We have also added information to this paragraph to related the categories back to the context of our study to help elucidate for the reading what the findings mean based on the results across these categories: “Thus, of the hotspots identified (n = 268 out of 5,702 city-wide walking-distance hexagons), 76.1% (n = 76.1) were not diminishing or sporadic, meaning they were sustaining or worsening over time.” |
|  | How did authors calculated the ‘average pit stop accessibility score’? needs to be detailed.   - We appreciate this feedback and the opportunity to make this analytical process clearer to readers. As described in a comment above, we have added a transition and made several edits to make the calculation process clearer to readers. |
| 192 | “average score of 0 for new hot spots”- is there any particular reason for 0 average? If yes then clarify.   - We appreciate this feedback and the opportunity to provide further clarification and context of these results to readers. We have added the following sentence to address this point: “As a result, all new hot spots as defined by the EHSA did not contain or have any adjacency to an area with a Pit Stop (i.e., an average Pit Stop Accessibility Score of 0), and that both persistent and diminishing hot spots had high average Pit Stop Accessibility Scores relative to all other hot spot categories identified.” |
|  | Maps should be detailed out. Figure 3 does not tell for which year it is prepared. What about the temporal changes? It is essential to produce temporal maps to see whether there are significant changes in spatial pattern of the subject matter.   - We are grateful for this feedback. We have added study years to the caption for Figure 3 to clarify that both maps reflect data from 2009 to 2022. |
|  | The result is presented well. However, critical arguments are missing and essential to produce.   - We are grateful for this reviewer’s careful read-through of our work, and we believe that in incorporating this feedback, the manuscript is much clearer and more accessible for an international audience. |
|  | There is an inconsistency in presentation of results. The PI Stop programme is likely to had a start in 2014 but the authors used data from 2009. How did the authors linked the temporal dataset?   - We appreciate this feedback. In order to examine possible effects of Pit Stop locations on 311 human/animal waste reports, we utilized data from years prior to the first Pit Stop installation. Pit Stops were introduced as a point layer into our maps with the year each was installed, and these results are summarized descriptively in Figure 2. The EHSA analysis was agnostic to Pit Stop locations, but are described relative to Pit Stop walking-distance hexagons in Table 4. |
|  | What are the arguments the analysis creating? Identify the main findings in bullet points at the end of the result section. Write the result section chronologically based on the aim/theme of the study.   - We are grateful for this feedback, and have created this section at the end of our Results section to incorporate it. |
|  |  |
| Discussion |  |
| 224-232 | This full paragraph is not suitable here. This is about the study area may be. Suggesting to transfer it to Intro or Method (study area sub section). Also, the paragraph does not have any importance with the study.   - We appreciate this feedback and have deleted the paragraph. |
| 234 | “this population is perhaps most affected”- how did authors claim this statement? What are the factors of ‘affected’?   - We are grateful for this feedback. We have changed our language to “especially vulnerable.” |
|  |  |
|  | The discussion section seriously lacks justification of arguments in comparison to the earlier studies. Authors are requested to revisit the discussion section. Authors can see “Mondal, S., Singh, D., & Kumar, R. (2022). Crime hotspot detection using statistical and geospatial methods: a case study of Pune City, Maharashtra, India. *GeoJournal*, *87*(6), 5287-5303.”   - We are grateful for this feedback and the opportunity to provide further justification. We have revised and expanded the Discussion section based on this comment and believe the writing and flow are much improved. |
|  | Start the discussion section by re-uttering the study objectives and how did the authors fulfil the objective(s). Then authors can discuss the main claims on the results. Re-write the section.   - We appreciate this feedback. We have now started the Discussion section with a restatement of our study aims and how we fulfilled them. |
|  |  |
| Limitations |  |
| 282 | “much of the city exhibited persistent, sporadic, or intensifying hotspot trends.”- I did not find any trend analysis or any map. Spatial unit of the study is neighbourhood. From where the unit ‘city’ come?   - We have edited this sentence to characterize walking-distance hexagons and neighborhoods. |
|  |  |
| Conclusion* |  |
|  | This is astonishing that the manuscript does not have ‘conclusion’. Authors are requested to include a conclusion section with specific policy implications and way forward. This is crucial.   - We appreciate this feedback. We have added a Conclusion section. |
|  | The study cited most of the google findings rather than actual scholarly articles. Please include scholarly articles where appropriate to theorize the idea and connect to the findings.   - We appreciate this feedback. We have added additional scholarly literature to the Discussion section. We have also removed some of the non-scholarly articles mentioned, in response to other reviewer comments. We believe the non-scholarly articles we have included are still relevant to the Discussion, as they describe additional efforts (henceforth, not yet evaluated by researchers) that have been implemented to reduce human waste on the streets of San Francisco, which we believe are important context for the future of this research for readers. |
|  | Only doing a hot spot analysis does not seem to be the primary objective here Authors need to bring arguments in line with urban health, sanitation practices, well-being, neighbourhood characteristics etc where possible. How will the result affect the decision-making process in USA?   - We are grateful for this feedback. We have added the Conclusion section to highlight how our results can inform future programs to increase access to sanitation facilities and pet waste reduction campaigns. |

Reviewer #3: The two most significant issues with this paper are identified by the authors in the limitation’s sections of their paper. However, I feel that they are underestimating the impact of these two factors which are that waste data in the 311 reports does not differentiate between human or animal waste and that restroom locations from Open Street Map have no temporal data. I feel that to be suitable for publication this paper needs to more conclusively demonstrate that animal waste is not a statistically significant portion of the 311 reports or demonstrate that variable does not affect the results. Not knowing the dates at which different rest room facilities became available is less of an issue but should also be addressed. Ideally the authors would demonstrate that the spatial patterning and availability of restrooms facilities has not changed in significant ways over the time period that this study examines, or changes would not affect the results. Two lesser issues are that some statements lack clear sourcing, and that data filtering and cleaning procedures are not explained, perhaps this latter point could be addressed through supplementary material. Otherwise, the analysis is sound, relevant, and of interest.

Other minor comments are as follows:
1) The 311 system should be explained in greater detail, particularly for international audiences who may not have had exposure to this system of reporting issues to civil government.

- We appreciate this feedback and the opportunity to make our manuscript more understandable for non-US, non-Canadian audiences. We have added additional information about how 311 systems are generally used, and provided a link to San Francisco’s website for their 311 system if readers would like to learn more.

2) There are a few statements that require additional sourcing (lines 46, 47).

- We appreciate the opportunity to correct this oversight. We have now provided sources for this sentence.

3) The referenced San Francisco neighborhoods should be displayed on a map as many audiences will not be familiar with this local geography.

- We appreciate this feedback and the opportunity to provide additional context for readers less familiar with the specific geography of San Francisco. Due to space limitations, we have added this map as Supplemental Figure, and referenced this figure in the Methods section.

4) Data cleaning and filtering operations should be described to allow for replicability of this analysis (line 87).

- We are grateful for this feedback. Another reviewer had a similar concern, and to address that concern, we created a table more clearly describing our data cleaning process. We now reference that table (presented just above this sentence) in the line mentioned here: “After cleaning and filtering the data as described in Table 2, we analyzed…”

5) Sourcing should be provided to justify the use of a 1.8 hectare walking distance hexagons (Iine 107).

- We appreciate this feedback. We have expanded our Methods section to include a description of the lack of consensus on appropriate walking distance to reach a restroom (along with several cited example recommendations that differ), and describe how we chose an intermediate distance between some of these recommended distances.

6) In line146 additional justification should be provided for why the specific 11 neighborhoods without Pit Stops were selected for including in the comparison in Figure 1.

- We appreciate this feedback. We have clarified in this sentence that the other 11 neighborhoods in the figure represent the top 11 neighborhoods without Pit Stops in terms of the number of 311 human/animal waste reports.

7) Lines 224-233 could more directly address the implications of comparing the Pit Stop program budget to other homelessness programs.

- Based on the feedback from another reviewer, we have deleted this paragraph.

8) I fail to see the relevance of lines 243-250, the connection of this paragraph to the rest of the paper should be elucidated or this section removed.

- We appreciate this feedback, and have removed this section.

9) I am unfamiliar with the “Karen effect” on line 260-262 and this should explained and sourced.

- We are grateful for this comment, and recognize that for an international audience, a “Karen” may not be common parlance. We have edited this section to described what we meant by unequal reporting bias without using this term: “It is possible, for example, that a small number of individuals living in specific parts of the city may make disproportionate use of the 311 system to report human or animal waste. the “Karen effect” involving Such massively unequal reporting behaviors could heavily skewed the waste report density in certain areas of the city, which may bias our findings.”

10) Fig. 3 would benefit from zooming in on the Northeast portion of the city which contains the hotspots.

- We appreciate this perspective. However, zooming into just the Northeast portion of the city could perhaps give the impression that that was the only area analyzed, or that the scope of the hotspots was larger than was observed. While we are grateful for this suggestion, we believe it is better to represent the entire city, as was analyzed.

Reviewer #4: This is a empirically solid paper! I commend the authors for providing a clearly-documented analytical approach, one which, to the extent possible, takes into account the obvious limitations to the study. (The coincidence of the pandemic with the study period is notable, as is the difficulty of controlling for the density of people experiencing homelessness.) I would propose several revisions, which should not present substantial barriers to incorporation.

First, I note the large number of hexagonal bins containing no reports of human or animal waste. This is a likely source of bias in the study's results, and could lead to either Type I or Type II errors. (Intuitively, I would also imagine that the lack of cold spots follows from the inflated zero count.) The authors should consider reporting results of an analysis in which these observations are dropped (potentially even including cases where there was only one report over the relatively long study period, which would seem to be a strong indicator that this is a case of dog-walkers more than unhoused people).

- We appreciate this thoughtful response. Type I errors in our analysis are unlikely because EHSA is conservative, and the areas with detected hot spots also contain high counts. Type II errors are possible, where true clustering is missed due to low counts across time, but we consider this an acceptable tradeoff for the high spatial resolution of the results which allows for a better connection with the Pit Stop locations to evaluate their influence.

Secondly, and relatedly, I think the analysis would be more compelling if the authors performed the analysis of only a subset of neighborhoods where there are known to be relatively large numbers of people experiencing homelessness. This would also have the effect of addressing the same zero-inflation bias as the above. With these first two suggestions, my basic point is that the study, as it stands, is a comparison of, essentially, areas where Pit Stop restrooms have been installed and all other areas in the city. The more compelling study is a comparison of areas where Pit Stop restrooms have been installed and areas experiencing at least broadly comparable levels of homelessness.

- We appreciate this feedback and the interest in our results. We believe the inclusion of the Pit Stop Accessibility Score should allow for the more focused comparison described above. Regarding the second point about a comparison of areas with comparable levels of homelessness that have either had Pit Stop restrooms installed relative to similar areas without Pit Stop restrooms installed, we agree this is a great idea, and could easily be the sole focus of a follow-up study and manuscript. We are grateful for this suggestion!

Finally, as an addition to the limitations section: in addition to disruptions brought about by the pandemic, I would think that, in many parts of the city, incidents of human waste would be substantially effected by changes in San Francisco's approach to policing homelessness, breakups of encampments, etc. (not to mention how it approaches street-cleaning). While this is hard to capture empirically, it's bears mentioning.

- We appreciate this comment, and completely agree. We have added a sentence to capture this, along with a citation.

Reviewer #5: The article shows high quality in terms of background introduction, research purpose, data processing and analysis results. However, there is still room for improvement in some aspects.

In the description of the research background, the literature review is relatively sufficient, but more discussion can be made on the international experience of similar urban health interventions to enhance the universality of the research. The uniqueness of San Francisco in the health issues of homeless people can be further emphasized, such as the special challenges and policies in homeless management and health facility provision in San Francisco compared with other cities.

- We have added some background on international barriers for public toilet usage, as well as some additional background and context on San Francisco specifically.

In addition, the previous research results and limitations of the Pit Stop project can be briefly reviewed to more clearly show the innovation and development of this study.

- We are grateful for this feedback and the opportunity to provide additional context to our readers. In response to this comment and another review comment, we have provided additional background in the introduction to address this point and better highlight the innovation of the present study.

The reported data may be limited by the reporting habits of residents (for example, some areas may have insufficient data due to fewer residents or low social attention). Although this is mentioned in the discussion section, it is recommended to add a description of the data limitations and possible coping strategies in the method section.

- We appreciate this feedback and have added a sentence in the Methods section to foreshadow the discussion of this issue.

The conclusion section is basically consistent with the research findings, but the core contributions of the article should be further summarized and policy recommendations should be highlighted. For example, it can be discussed how the Pit Stop project can optimize site selection or improve service efficiency to enhance the practical value of the research.

- We are grateful for this feedback and the opportunity to incorporate this suggestion into our manuscript. We have added this idea into our Conclusion section.

When analyzing the results, further exploration could be conducted into why improvements were seen in some areas (such as the Tenderloin) but not in others. For example, differences between areas could be considered, such as homeless population density, program coverage, and community engagement.

- We appreciate this comment and the suggestions for future work. We believe all of these factors could be relevant to Pit Stop usage and 311 reports of waste, and we are very interested in pursuing analysis of homeless population density, community engagement/perception in a future study. In our current manuscript, we believe the Pit Stop Accessibility Score addresses the program coverage or intensity by using the density and duration of sites in its calculation, such that higher scores should indicate greater program coverage for that area.

Overall, the study has high publication value and is recommended for publication after revision.

Reviewer #6: Review summary

The manuscript involves an Emerging Hot Spot Analysis of 311 reports for human/animal waste in San Francisco, between 2009 and 2022 building upon the Pit Stop Program and previous work that has been conducted concerning the analysis of these spots, both spatially and temporally. The authors state the problem successfully, presenting the situation in San Francisco compared to other areas of the USA and explain the Pit Stop Program as well how the 311 reports are collected. The research gap is not fully described and needs further explanation. The methods used in the manuscript are well described and the methods used are appropriate. There are some minor issues with the description of data as well as the clarification of categories of hot spots used in the research work. The results are presented in a sophisticated way and the subject has been approached quite well. The use of graphs and table is helpful and informative. The results present some a few minor issues. The discussion are developed in an holistic way, including points that have not been mentioned before in the previous sections, such the “medical waste”. It is important that the limitations section has been thoroughly described. The map and graphs presented are very well made. It is important the authors have added supplementary material. They have not added the full list of reports used but this is not a big issue as these data are publicly available, as it is mentioned in the manuscript. According to my opinion, the manuscript can be published after minor changes.

Major issues

In my opinion, there are no major issues concerning the manuscript.

Minor issues

1. The research gap that can be covered by the manuscript should be better developed.

- We appreciate this feedback and the opportunity to better identify the unique contribution of our study to the field. In response to this comment and other reviewer feedback, we have added several sentences to our Introduction to highlight what is known and what the current gap in the literature is that our study fills.

2. Probably the exact number of neighborhoods can be mentioned (line 97).

- We appreciate this feedback, and have added the total number of neighborhoods to this sentence.

3. How was the comfortable walking distance to a restroom in time calculated? If it comes from a published work then this can be mentioned.

- We are grateful for this comment and the opportunity to provide readers with more context regarding this analytical decision. We have added the following sentences with citations to our Data Analysis section where an appropriate walking distance is first described: “No universal standard for a comfortable or appropriate walking distance to restrooms exists, although multiple recommendations have been suggested by different organizational bodies around the world for different situations and stakeholders, including 60 meters or ~0.9 hectares (39), 100 meters or ~2.6 hectares (40), and 200 feet or ~0.9 hectares (41). Given the lack of consensus, we selected an intermediate distance of 1.8 hectares.”

4. It would be better if the categories of spots (lines 111, 112) are described, even with an example.

- We are grateful for this feedback and the opportunity to provide more clarity to readers. We have expanded and moved the definitions of the categories in the table that is in the Results section up here, to provide that specificity to readers about the categories when we introduce what the EHSA analysis is.

5. The description of neighborhood results (lines 131-138) is a bit vague, making it difficult to follow.

- We appreciate this feedback and the opportunity to clarify. We have edited this particular paragraph based on this comment and other comments made by other reviewers to improve readability and clarity.

6. Even at the end of the results section, the connection between pit stop points and neighborhoods was unclear, according to my opinion.

- We are grateful for this feedback. We have edited the Results section based this comment and others from additional reviewers and believe that the connection between Pit Stops and neighborhoods is now clearer.

7. Even though I find the maps used very presentable, informative and clear, I would suggest their separation in two different maps as they are not very well visible in one page.

- We appreciate this feedback. We debated this point on our team as well, and decided on the side-by-side presentation to facilitate visual comparison between waste report density (left) and EHSA categories (right), which would be difficult to do on separate pages. We believe the ability to more directly compare the 2 maps is a benefit that outweighs the cost in reduction in size.

8. Table 1 is clear and well made. However, it was unclear to me how the average pit stop accessibility score was used later in the manuscript. It seems that it is not sufficiently developed although it is a very useful information.

- We appreciate this feedback, and have expanded our write-up of Pit Stop Accessibility scores and what they mean toward the end of the Results section.

9. It would be probably useful for the “medical waste” section to be described in the methods section and not be presented for the first time in the discussions.

- We are grateful for this comment, and have already removed this section based on feedback from another reviewer.

10. Concerning the limitations, they are very well developed. Nevertheless, could the authors describe any actions taken concerning these limitations? It is not clear to me but this is a section that probably not many actions can be taken anyway.

- We appreciate this feedback, and have expanded our Limitations section to highlight how these issues could possibly be addressed.

11. The full list of reports and data could be presented but as the authors mentioned these data are open and accessible so this is not a problem.

- Thank you for this comment. We believe the table formatting of our inclusion/exclusion criteria should also facilitate others replicating our work.

12. The supplementary graph is very clear and informative. I would recommend to be added in the main body of the manuscript but this is just a recommendation.

- We have added the supplemental figure to the main body of the manuscript, and a short paragraph describing the results.

13. It would be really great if the readers could read a section on how this study and generally studies like this can contribute to better implementation of Pit Stop Programs.

- We appreciate this comment, and recognize the need to make scientific findings highly relevant for decision-makers. We have expanded our Conclusion section to help describe how our results might be used in this way (with the caveat that our analysis is not causal, and thus we cannot say for sure that installation a Pit Stop location will lead to a particular effect).

Positive issues

• This is a research work with a detailed spatial analysis, useful for both scientific and social policy issues.
• The maps and graphs of the manuscript are created in a sophisticated way using R and GIS, adding up to the quality of the work.
• The introduction section might not lengthy but it contains everything that is needed for the reader to understand what is included in the research (the selection of San Francisco, the 311 reporting, etc.), only lacking the gap description.
• The description of the selection of reports and data is clear and well developed.
• Both the spatial and temporal analysis are very well approached, as well as neighborhood comparisons, despite some points being unclear.
• Limitations are very well described.

- We appreciate this feedback!
